# Rapid Weight Loss Practices in Grapplers Competing in Combat Sports

**DOI:** 10.3389/fphys.2022.842992

**Published:** 2022-02-09

**Authors:** Marijana Ranisavljev, Jovan Kuzmanovic, Nikola Todorovic, Roberto Roklicer, Milorad Dokmanac, Mario Baic, Valdemar Stajer, Sergej M. Ostojic, Patrik Drid

**Affiliations:** ^1^Faculty of Sport and Physical Education, University of Novi Sad, Novi Sad, Serbia; ^2^Belgrade Sports Academy, Belgrade, Serbia; ^3^Faculty of Kinesiology, University of Zagreb, Zagreb, Croatia

**Keywords:** rapid weight loss, grappling, combat sports, weight loss, weight class

## Abstract

**Background:**

Grappling is a wrestling style that combines different techniques such as freestyle wrestling, jiu-jitsu, judo, sambo, and others. As with other combat sports, it requires categorizing the athletes in weight classes, which leads to the use of certain methods to lose body weight in a short amount of time which poses a serious threat to athletes’ health and wellbeing. Therefore, the objective of this study was to investigate the most widespread rapid weight loss (RWL) methods and sources of influence used by grappling athletes.

**Methods:**

A total of 145 athletes took part in the study by voluntarily filling out a questionnaire regarding their weight loss techniques and methods. They were divided into two groups, male (27.7 ± 5.2 years, 1.76 ± 0.13 m, and 82.1 ± 20 kg) and female (27.33 ± 6.3 years, 1.65 ± 0.08 m, and 64.3 ± 10.4 kg), for further statistical analysis. After calculating descriptive statistics for all the variables, a *t*-test was conducted for gender differences in weight loss and weight regain, and a chi-square test measured the diversity in techniques and source of influence.

**Results:**

Out of 145 participants, 120 athletes (85.2%) reported engaging in rapid weight loss prior to weigh-in. Coaches (52.4 and 59%) and teammates (42.6 and 22.1%) seemed to be the most influential on their rapid weight-loss strategies, whereas physicians (17.1 and 17.9%) and parents (23.2 and 23.1%) were the least influential. A statistical difference between men and women (*p* = 0.05) was found when teammates were a source of influence (42.6 and 21.1%, respectively). Regarding the methods used, both groups practiced gradual dieting (85.4 and 79.5%) as the most prevalent, followed by increased exercise (79 and 66.6%) and sauna (78.7 and 66.6%). Moreover, men trained in plastic suits significantly more often than women (67.1 vs. 41%, *p* = 0.01).

**Conclusion:**

Rapid weight loss is detrimental to athletes’ health and wellbeing. Hence, it is crucial to find and implement methods that will control and ultimately limit its use in combat sports. Physicians and nutritionists need to be closely linked with the staff, collaborate and supervise the weight cutting.

## Introduction

Rapid weight loss (RWL) is characterized by temporary weight loss of at least 5% of body weight, mainly 2–3 days before the weigh-in (Artioli et al., 2016). It is highly prevalent among combat sports where athletes tend to compete at the lightest weight possible, believing that it will provide an advantage over their opponent (Gann et al., 2015). Reports from previous studies (Artioli et al., 2010b; Brito et al., 2012) indicate that RWL is achieved through a combination of aggressive and harmful methods, highly prevalent among competitors (Artioli et al., 2016). Certainly, the most common methods for RWL practices include fluid restriction and dehydration, long periods of fasting, and high-intensity exercise in plastic suits (Brito et al., 2012). Coming from the perspective that prioritizes athletes’ health, several health-related issues are linked to RWL, including acute and chronic effects such as hypohydration and reduced plasma volume (Reljic et al., 2013), which lead to the increased risk of cardiovascular problems (González-Alonso et al., 1997). Moreover, RWL can result in a hormonal imbalance (Roemmich and Sinning, 1997), bone loss (Prouteau et al., 2006), and suppression of immune function (Suzuki et al., 2003). RWL can jeopardize athletes’ health, primarily affecting the increase of specific blood biomarkers and psychological parameters, such as tension, anxiety, and depression (Roemmich and Sinning, 1997). In 1997 three wrestlers experienced cardiorespiratory and thermoregulatory complications while preparing for a competition which led to a fatal outcome due to practicing RWL techniques (Centers for Disease Control and Prevention [CDCP], 1998). Also, two MMA fighters died between 2013 and 2016 on similar occasions (Crighton et al., 2016). These lead to certain rule changes, but RWL is still one of the biggest problems in sports with weight categories.

The magnitude of RWL among sports may be determined by the type of sport, competition structure (one match event vs. tournament), and duration between weigh-in and competition (Matthews et al., 2019). For example, it was reported recently that 87% of sambo athletes cut their weight before the competition (Drid et al., 2021), and almost 90% of female sambo athletes reported doing the same (Todorovic et al., 2021). Wrestling is the most commonly studied sport for RWL, with the prevalence of RWL among wrestlers varying between 40 and 90% (Khodaee et al., 2015). However, wrestling has several different styles, and all of these differences among the styles can be viewed as a sport on its own. For instance, grappling is a wrestling style that combines techniques originated in different sports such as Freestyle Wrestling, Brazilian Jiu-Jitsu, Judo, Sambo. Grappling is divided into two styles: Gi, where athletes wear a kimono, and No-Gi, where athletes wear shorts and a compression shirt (United World Wrestling [UWW], 2021). The aim of grappling is to take down, control, and submit the opponent on the ground (Stajer and Marinkovic, 2013). Interestingly, because most of the fight time wrestlers spend on the ground, it can be considered one of the safest wrestling sports to practice (United World Wrestling [UWW], 2021).

Based on the authors’ knowledge, no previous studies evaluated the magnitude of RWL among grappling athletes. Therefore, this study aimed to identify the methodologies of RWL, with the central hypothesis set to determine the most widespread methods and sources of influence used by grappling athletes and to determine any differences in RWL strategies among genders.

## Materials and Methods

### Data Assessment

To evaluate RWL methods among male and female wrestlers, we adopted a standardized and validated RWL questionnaire developed by Artioli et al. (2010c). This self-reporting instrument designed to assess RWL patterns consists of 21 items relating to personal information, competitive level, nutrition status, RWL history, and RWL behaviors. To better assess and evaluate RWL magnitude among athletes, the questionnaires were translated from the original Portuguese language to several languages (e.g., Russian, Italian, Spanish, French, Serbian, Romanian, German, and Bulgarian) to facilitate data collection. Thus far, the questionnaire was validated in Portuguese and French only (Pélissier et al., 2021). All of the questionnaires were anonymous. Also, investigators were available to athletes to provide detailed information about the questionnaires and answer any questions during the procedure if necessary.

### Subjects

**FIGURE 1:**
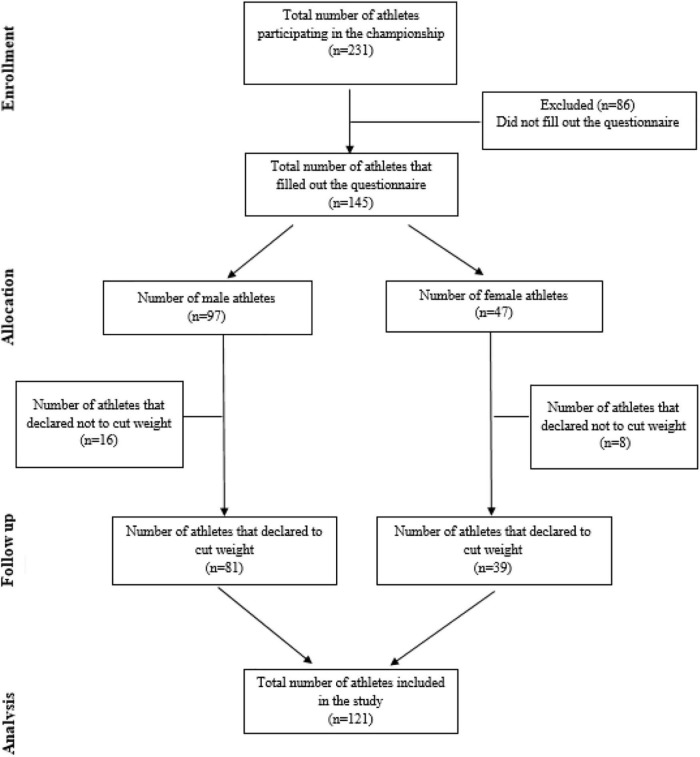
Flow-chart of participants selection.

Participants in the study were top-level athletes competing at the World Wrestling Championship in Belgrade, Serbia. A total of 231 athletes from 22 countries were competing in the tournament. Of these, 145 participants from 16 countries completed the questionnaires: Ukraine (18.5%), Russia (17.1%), Kazakhstan (11.6%), United States (7.5%), Kyrgyzstan (6.2%), Italy (6.2%), Spain (5.5%), France (5.5%), India (4.1%), Serbia (4.1%), Romania (3.4%), Moldova (2.7%), Armenia (2.7%), Germany (2.1%), Belorussia (1.4%), and Bulgaria (1.4%). They were further divided into two groups: male athletes (27.7 ± 5.2 years, 1.76 ± 0.13 m, and 82.1 ± 20 kg) and female athletes (27.33 ± 6.3 years, 1.65 ± 0.08 m, and 64.3 ± 10.4 kg). The selection of participants is displayed in detail in Figure 1. The study was conducted according to the Helsinki Declaration (Hellmann et al., 2014) and obtained ethical approval from the Ethical Committee of the University of Novi Sad, Serbia (Ref. No. 46-06-02/2020-1). The investigators provided an extensive explanation of the purpose, objectives, and goals of the study, and all of the athletes gave written informed consent to their voluntary participation in the study.

### Statistical Analysis

Collected data were analyzed using SPSS statistical software (ver. 24.0) (IBM Statistics, Armonk, NY, United States). First, data were checked for normality using a Kolmogorov–Smirnov test. Descriptive statistics were calculated for all the variables involved in the analysis, including height, weight, RWL frequency, RWL methodology, and influence in weight-cutting practices. Then, gender differences regarding weight loss and weight regain were evaluated through a *t*-test, while diversity in RWL techniques and source of influence was calculated through the Chi-square test. The significance level was set at *p* = 0.05.

## Results

From a total sample of 145 athletes, 120 athletes (82.5%) reported cutting their weight before competitions. Athletes usually start cutting weight 9 days before the competition and they normally cut approximately 3.9 and 3 kg, for men and women, respectively. They reported starting their RWL practices at 19 years of age. There were no statistical differences between genders in information concerning cutting weight history (see Table 1).

**TABLE 1:** Rapid weight loss (RWL) history.

	Group	Mean	SD	*t*	*P*
How much weight do you usually cut before the competition? (kg)	Male	3.8	2.5	1.36	0.18
	Female	3.1	2.5		
How many days before the competition do you usually cut weight? (days)	Male	10.9	12.6	0.43	0.67
	Female	9.91	7.5		
At what age did you start to cut weight before the competition? (years.)	Male	19	5.2	−0.55	0.58
	Female	19.7	2.7		
How much weight do you usually regain after the competition? (kg)	Male	3.5	2.3	1.74	0.08
	Female	2.9	1.3		
At what age did you begin to compete practice wrestling?	Male	14.1	6.1	−1.3	0.18
	Female	15.9	7.8		
Score obtained in RWL questionnaire	Male	29.1	2.3	0.66	−0.44
	Female	30.3	1.7		

*SD, standard deviation; t, independent t-test; p, probability.*

The most significant influence in RWL strategies on athletes had coaches (52.4 and 59%) and teammates (42.6 and 22.1%). In comparison, physicians (17.1 and 17.9%) and parents (23.2 and 23.1%) had less influence. The significance was determined computed as the sum of the answers “somehow influential” and “very influential.” The statistically significant difference between genders was found in the variable influence of teammates (42.6 vs. 21.1%, *p* = 0.05) (see detailed in Table 2).

**TABLE 2:** Source of influence.

Source of influence	Gender	Not influential	Little influential	Unsure	Somehow influential	Very influential	χ^2^	*P*
Teammate	Male	28 (34.1%)	15 (18.3%)	4 (4.9%)	23 (28%)	12 (14.6%)	9.45	**0.05**
	Female	15 (38.5%)	7 (17.6%)	8 (20.5%)	6 (15.4%)	3 (7.7%)		
Fellow wrestler	Male	34 (41.5%)	12 (14.6%)	11 (13.4%)	14 (17.1%)	11 (13.4%)	0.85	0.93
	Female	14 (35.9%)	8 (20.5%)	5 (12.8%)	6 (15.4%)	6 (15.4%)		
Physician	Male	49 (59.8%)	11 (13.4%)	8 (9.8%)	5 (6.1%)	9 (11%)	3.21	0.52
	Female	12 (64.1%)	5 (12.8%)	2 (5.1%)	5 (12.8%)	2 (5.1%)		
Personal trainer	Male	41 (50%)	14 (17.1%)	5 (6.1%)	10 (12.2%)	12 (14.6%)	2.99	0.56
	Female	20 (51.3%)	5 (12.8%)	5 (12.8%)	6 (15.4%)	3 (7.7%)		
Coach	Male	20 (24.4%)	15 (18.3%)	4 (4.9%)	21 (25.6%)	22 (26.8%)	2.13	0.71
	Female	8 (20.5%)	5 (18.2%)	3 (7.7%)	14 (35.9%)	9 (23.1%)		
Parents	Male	45 (54.9%)	10 (12.2%)	8 (9.8%)	5 (6.1%)	14 (17.1%)	4.75	0.32
	Female	23 (59%)	3 (7.7%)	4 (10.3%)	6 (15.4%)	3 (7.7%)		
Dietitian	Male	45 (54.9%)	12 (14.6%)	3 (3.7%)	11 (13.4%)	11 (13.4%)	3.31	0.51
	Female	22 (56.4%)	3 (7.7%)	4 (10.3%)	4 (10.3%)	6 (15.4%)		

*The bolded values are meant to draw the reader’s attention to statistical significance.*

Regarding the methods athletes applied during weight cutting, there were statistical differences between male and female athletes in variable training in the plastic suits (67.1 vs. 41%, *p* = 0.01), respectively. The most commonly used methods by both groups combined for RWL (calculated as the sum of the answers “always” and “sometimes”) were gradual dieting (85.4 and 79.5%), followed by increased exercise (79 and 66.6%), and sauna (78.7 and 66.6%). In contrast, the least frequent methods were diet pills (8.5 and 10.2%), followed by vomiting (8.5 and 12.8%), and diuretics (8.5 and 15.4%) for male and female athletes, respectively (see detailed in Table 3).

**TABLE 3:** Reported methods used by athletes during RWL.

Source of influence	Gender	Always	Sometimes	Rarely	Never	Do not use it anymore	χ^2^	*P*
Gradual dieting	Male	41 (50%)	29 (35.4%)	3 (3.7%)	4 (4.9%)	5 (6.1%)	1.4	0.85
	Female	20 (51.3%)	11 (28.2%)	3 (7.7%)	2 (5.1%)	3 (7.7%)		
Skipping meals	Male	8 (9.8%)	42 (51.2%)	9 (11%)	17 (20.7%)	6 (7.3%)	4.2	0.38
	Female	6 (15.4%)	16 (41%)	8 (20.5%)	5 (12.8%)	4 (10.3%)		
Fasting	Male	7 (8.5%)	26 (31.7%)	16 (19.5%)	28 (34.1%)	5 (6.1%)	3.63	0.46
	Female	2 (5.1%)	13 (33.3%)	5 (12.8%)	13 (33.3%)	6 (15.4%)		
Restricting fluid ingestion	Male	13 (15.9%)	33 (40.2%)	12 (14.6%)	19 (23.2%)	5 (6.1%)	0.72	0.95
	Female	6 (15.4%)	13 (33.3%)	6 (15.4%)	11 (28.2%)	3 (7.7%)		
Increased exercise	Male	33 (40.2%)	31 (37.8%)	6 (7.3%)	6 (7.3%)	6 (7.3%)	3.73	0.44
	Female	13 (33.3%)	13 (33.3%)	7 (17.9%)	4 (10.3%)	2 (5.1%)		
Training in heated room	Male	14 (17.1%)	41 (50%)	8 (9.8%)	15 (18.3%)	4 (4.9%)	13.24	**0.01**
	Female	6 (15.4%)	10 (25.6%)	11 (28.2%)	12 (30.8%)	0 (0%)		
Sauna	Male	15 (18.3%)	49 (59.8%)	8 (9.8%)	7 (8.5%)	3 (3.7%)	2.1	0.72
	Female	7 (17.9%)	19 (48.7%)	5 (12.8%)	6 (15.4%)	2 (5.1%)		
Training in plastic suits	Male	8 (9.8%)	25 (30.5%)	15 (18.3%)	29 (35.4%)	5 (6.1%)	4.41	0.35
	Female	2 (5.1%)	19 (48.7%)	5 (12.8%)	12 (30.8%)	1 (2.6%)		
Use plastic suit all day	Male	3 (3.7%)	21 (25.6%)	15 (18.3%)	36 (43.9%)	7 (8.5%)	2.58	0.63
	Female	1 (2.6%)	9 (23.1%)	6 (15.4%)	22 (56.4%)	1 (2.6%)		
Spitting	Male	5 (6.1%)	28 (34.1%)	13 (15.9%)	30 (36.6%)	6 (7.3%)	3.67	0.45
	Female	2 (5.1%)	12 (30.8%)	7 (17.9%)	18 (46.2%)	0 (0%)		
Laxative	Male	2 (2.5%)	10 (12.3%)	5 (6.2%)	60 (74.1%)	4 (4.9%)	3.53	0.47
	Female	0 (0%)	9 (23.1%)	3 (7.7%)	26 (66.7%)	1 (2.6%)		
Diuretics	Male	1 (1.2%)	6 (7.3%)	5 (6.1%)	66 (80.5%)	4 (4.9%)	1.34	0.85
	Female	1 (2.6%)	5 (12.8%)	2 (5.1%)	29 (74.4%)	2 (5.1%)		
Diet pills	Male	0 (0%)	7 (8.5%)	5 (6.1%)	66 (80.5%)	4 (4.9%)	7.33	0.12
	Female	2 (5.1%)	2 (5.1%)	1 (2.6%)	34 (87.2%)	0 (0%)		
Vomiting	Male	0 (0%)	7 (8.5%)	3 (3.7%)	66 (80.5%)	6 (7.3%)	6.74	0.15
	Female	2 (5.1%)	3 (7.7%)	0 (0%)	33 (84.6%)	1 (2.6%)		

*The bolded values are meant to draw the reader’s attention to statistical significance.*

## Discussion

The main aim of this study was to evaluate prevalence, methods, and sources of influence among grapplers. The overall prevalence of RWL practices was 82.5%. Although we found differences between male and female athletes, the overall risk of RWL on health was equal (29.1 vs. 30.3), respectively. Similar values were obtained for international and top-level athletes in the study of Artioli et al. (2010b). The RWL presents one of the most serious problems in combat sports, and it is essential to examine methods and sources of influence among athletes. The first notable difference between genders was observed in the source of influence. Male athletes shared experiences between themselves more often than female athletes (42.6 vs. 21.1%, *p* = 0.05). A possible explanation is in the psychological fact that women do not want to talk too much about weight, and so it is possible that they did not want to discuss potential practices for losing weight (Lieberman et al., 2012). Coaches were the most influential people to all athletes, regardless of gender, while parents and physicians were the least influential. This deviates to some extent from previous studies, especially in terms of parental influence (Kons et al., 2020; Drid et al., 2021; Figlioli et al., 2021; Todorovic et al., 2021). It is possible that the athletes in our study were older, and therefore there were not influenced by their parents. Another problem is the weak influence of doctors and nutritionists among athletes. The weight loss and recovery process should be under the supervision of professional staff, nutritionists, and physicians to protect athletes’ health and performance.

One of the surprising results was the age at which they started to cut weight before the competition. Athletes in our sample started their RWL practices as adults (19 years old). These results, however, are different from results obtained in previous studies (Berkovich et al., 2016; Kons et al., 2020; Drid et al., 2021; Figlioli et al., 2021; Todorovic et al., 2021), while some reports indicate that athletes even as young as 5 years old lost weight to compete (Sansone and Sawyer, 2005). A possible explanation may be in the fact that our respondents started to train grappling in later years, compared to athletes from the mentioned studies.

The most used methods in this study were gradual dieting, sauna, and skipping meals. Comparable results were found in the sample of elite male and female Sambo athletes (Figlioli et al., 2021; Todorovic et al., 2021). In one small sample of judo athletes, the magnitude of RWL was lower than in this study, while the methods they used were predominantly gradual dieting and increasing exercise (Gann et al., 2015; Kons et al., 2020). A recent publication from Lakicevic et al. (2021a) discussed this issue in detail. Second, a statistically significant difference is that male athletes more often trained in heated rooms compared to female athletes (67.1 vs. 41%, *p* = 0.01). On the other hand, women trained in plastic suits more compared to men (53.8 vs. 40.3%), respectively. For example, in the study by Drid et al. (2021), no differences were shown between genders in their study, both in absolute or relative terms. Although previously mentioned practices may be less harmful, a certain number of athletes reported the usage of laxatives, diuretics, and diet pills. These results are worrisome, not because they represent prohibited substances by the World Anti-Doping Agency (Cadwallader et al., 2010), but because of the adverse outcomes on the athletes’ health (Wile, 2012). In addition, in one study, it was reported that 72% of high school wrestlers engaged in at least one potentially harmful RWL method (Kiningham and Gorenflo, 2001).

We are aware that RWL is a serious problem, which can be approached by trying to change things in a planned way or radically change the rules. In the past, there have been initiatives to limit the abuse of RWL, such as the introduction of hydration tests, measurements at the beginning of the season to determine the weight category of athletes and limit weight loss to a maximum of 1.5% of body weight per week (Artioli et al., 2010a). What is certain is that RWL is extremely harmful to the health of athletes. Several authors outlined the potential medical consequences of RWL, both acute and long-term (Oppliger et al., 1996; Turocy et al., 2011). Particular concerns have been raised in cases where athletes lose more than 5% of their body mass, mainly due to dehydration (Khodaee et al., 2015). Dehydration can lead to decreased plasma volume, further resulting in increased heart rate and decreased stroke volume (Watanabe et al., 2020). Moreover, dehydration causes acute kidney damage and can further lead to adverse events in other body systems (Lakicevic et al., 2021b). In these states, athletes are more susceptible to heat injury (Ozkan and Ibrahim, 2016). In addition to these results, Montani et al. (2015) found a correlation between frequent weight loss cycles and cardiovascular disease and type 2 diabetes among the general population. Weight loss can negatively affect the immune system and increase illness possibilities by significantly increasing pathogenic activity (Kowatari et al., 2001). Also, it is essential to highlight the cognitive and behavior changes during and after RWL, i.e., wrestlers often felt angry while losing weight (Sundgot-Borgen and Garthe, 2011). Moreover, this can be seen as a positive reaction, but these angry states are often followed by a decrease in memory and concentration (Franchini et al., 2012), mood changes (Choma et al., 1998), and eating disorder (Davis et al., 2002). The question is, at what cost and why do athletes practice these RWL strategies, widely proven to be dangerous to their health? According to previous research, the results are ambiguous, where some studies show no changes of RWL on performance (Serfass et al., 1984; Webster et al., 1990), where others show a decrease in performance (Filaire et al., 2001; Hall and Lane, 2001). In the ACSM consensus statement, existing literature was reviewed in detail, and it was concluded that RWL negatively affects performance and health, and this problem should be addressed based on the specific characteristics of each sport (Burke et al., 2021). Based on everything else, it is time to open the question and discuss the possibility of banning RWL in combat sports.

The current study has several limitations. First, the methodology lacks an experimental approach. This was a cross-sectional study where we conducted an informative questionnaire, with no further evaluation of body composition and blood and urine biomarkers. As a second limitation, we can mention the sincerity of the respondents when filling out the questionnaire, especially in the sections for some dangerous or prohibited methods, even though the questionnaires were anonymous. Also, some of the respondents did not understand all the questions best due to language barriers, although our researchers tried to help them when necessary. Finally, the respondents were not monitored before and after RWL. Future research should be based on detailed monitoring of competitors during weight loss to determine as accurately as possible the consequences and harmfulness of RWL methods.

## Conclusion

This is the first study to examine the effects of rapid weight loss in grapplers. One of the most significant advantages of this study is that our sample is the most representative possible, since all of our participants are international competitors. The results we obtained in this population of athletes do not differ much from those obtained in research on sambo wrestlers, judokas, and wrestlers. However, representation and methods in both genders are a cause for concern. It is necessary to prioritize athletes’ health over success. Potential solutions are to shorten the time between weighing and fighting, as well as adding hydration level tests, and determining the weight category that the athlete must not cross at the beginning of the competition season. In addition, it is essential that weight loss and recovery processes are carried out under the supervision of professional staff, nutritionists, and doctors to protect athletes’ wellbeing and performance. Educating coaches and hiring nutritionists in teams would in many ways enable healthier body mass loss among combat sports athletes.

## Data Availability Statement

The raw data supporting the conclusions of this article will be made available by the authors, without undue reservation.

## Ethics Statement

The studies involving human participants were reviewed and approved, and the study was conducted according to the Helsinki Declaration and obtained ethical approval from the Ethical Committee of the University of Novi Sad, Serbia (Ref. No. 46- 06-02/2020-1).

## Author Contributions

MR, NT, VS, SO, and PD were involved in study conception and design and wrote the first draft of the manuscript. JK, RR, MD, and MB collected the data and analyzed the data. All authors revised, edited, and approved the final manuscript.

## Conflict of Interest

The authors declare that the research was conducted in the absence of any commercial or financial relationships that could be construed as a potential conflict of interest.

## Publisher’s Note

All claims expressed in this article are solely those of the authors and do not necessarily represent those of their affiliated organizations, or those of the publisher, the editors and the reviewers. Any product that may be evaluated in this article, or claim that may be made by its manufacturer, is not guaranteed or endorsed by the publisher.
